# Chitosan Gauze in the Management of Acute Postpartum Hemorrhage in a Mexican Third-Level Institution: A Case Report

**DOI:** 10.7759/cureus.71079

**Published:** 2024-10-08

**Authors:** Roberto Velasco Sordo, Humberto López Maldonado, Daniel A. Ramirez Flores, Eduardo Ibarrola Buen Abad, Pablo Vilchis Nava

**Affiliations:** 1 Obstetrics and Gynecology, Centro Médico American British Cowdray (ABC), Mexico City, MEX

**Keywords:** chitosan gauze, hysterectomy, intrauterine balloon tamponade, obstetric complications, postpartum hemorrhage, uterine packing

## Abstract

Obstetric hemorrhage is the main cause of maternal death worldwide; over the years, its management has been based on uterotonic drugs as well as definitive and non-definitive surgical techniques. We report the case of a patient with multiple risk factors for obstetric hemorrhage who was given the classic management for postpartum hemorrhage before the addition of chitosan gauze, which resulted in adequate remission of the condition. The patient had an adequate in-hospital evolution; the chitosan gauze was removed after 24 hours, and she was discharged after 48 hours without active bleeding. When identifying women at high risk of obstetric hemorrhage, having chitosan gauze in the operating room could be a point of good care.

## Introduction

The American College of Gynecology and Obstetrics defines postpartum hemorrhage (PPH) as blood loss greater than or equal to 1000 mL, or blood loss accompanied by signs and symptoms of hypovolemia in the first 24 hours after birth regardless of the route of delivery [1].

PPH represents the main cause of maternal death, causing 127,000 deaths per year worldwide [2]. When evaluating a patient who is bleeding, it is helpful to consider the “Four T’s” mnemonic, which stands for tone, trauma, tissue, and thrombin [3]. The major risk factors for PPH are the spectrum of placenta accreta, known coagulation defects, and history of PPH [4].

From 1980 to the present, mortality due to PPH has decreased as a result of the implementation of uterotonic drugs, massive transfusion protocols, and advanced surgical techniques [5].

As an alternative to management, in 2012 the use of a chitosan hemostatic gauze was described for the first time by Schmid and colleagues, who described the case of a patient who underwent an elective cesarean delivery at a gestational age of 37 weeks for complete placenta previa without evidence of placenta accreta. Two hours after the uneventfully completed initial surgery, heavy vaginal bleeding was observed, which was controlled with uterotonics. Two hours after that, a new bleeding episode started. As curettage revealed no retained placental tissue; it was decided to perform a relaparotomy. After B-Lynch sutures proved ineffective, uterovaginal packing was performed with chitosan-covered gauze. Hemostasis was achieved, and the chitosan gauze was left in the uterus for 36 hours. After the removal of the chitosan gauze, no more bleeding occurred [6].

The main component of chitosan gauze is chitin, a polysaccharide that is part of the exoskeleton of the crustacean Pandalus borealis. In order to produce the chitosan gauze, chitin must be biochemically modified through two reactions. In the first reaction, chitin is deacetylated to obtain chitosan. Subsequently, in the second reaction, the amino group of chitosan is protonated, and a modified chitosan is obtained, with the protonated amino group giving it its unique mechanism of action [7-9].

Chitosan gauze works through two major mechanisms: first, it generates mechanical compression towards the uterine walls. Second, the cationic charge that chitosan obtains when modified allows the formation of electrostatic complexes comprising negatively charged red blood cells, thus activating coagulation through an alternative route [10].

Products containing chitosan do not cause allergic reactions in people with shrimp allergies. Kirk and colleagues hypothesized that subjects who are allergic to shrimp may not experience a reaction to chitosan due to the extraction process. During extraction of the shellfish exoskeleton, acid destroys carbohydrate side chains, removing proteins such as tropomyosin, which are responsible for allergic reactions [11].

## Case presentation

In February 2024, a 31-year-old woman, gravida 7, para 3, abortion 3, presented to the Labor and Delivery Department at 38 weeks and seven days of gestational age for labor induction. She had no relevant family history. Her past medical records showed a sleeve gastrectomy in 2011, a flexible ureteroscopy in 2020, and a hysteroscopy in 2023, all of which were reported without complications. The patient admitted to smoking (three cigarettes per day for 15 years) and social alcohol consumption, both of which were suspended during pregnancy.

The patient was diagnosed with gestational hypertension at 36.6 weeks of gestation and was managed with alpha-methyldopa 500 mg every eight hours and nifedipine 30 mg every 12 hours, achieving adequate blood pressure control. The last obstetric ultrasound at 36 weeks of gestation indicated a single live fetus weighing 2700 g, in cephalic presentation, with a normal amniotic fluid index and no placental or umbilical cord abnormalities.

Upon admission, physical examination demonstrated adequate pelvimetry, a category-one cardiotocographic registry, and a Bishop score of six. Labor induction was conducted with oxytocin for eight hours. The latent phase lasted five hours, and the active phase (>5 cm dilation) lasted three hours. When the fetal station was +4, the patient was transferred to the delivery room. The delivery was performed using Simpson forceps due to arrest on the fetal descent without complications, resulting in a vigorous fetus with an appearance, pulse, grimace, activity, and respiration (APGAR) score of 9/9.

Post-delivery, active management of the third stage of labor was achieved, and a routine manual uterine inspection was performed, during which increased bleeding secondary to uterine atony was noted. Subsequently, uterotonic agents, including carbetocin and ergometrine, were administered, with no visible response. Therefore, it was decided to perform an obstetric curettage with uterine artery clamping using Zea Prado. Despite these interventions, hemorrhage persisted. Therefore, a Foerster clamp was placed in the anterior cervical lip, and we decided to pack the uterine cavity with chitosan gauze, and the active bleeding ceased. As a preventive measure, 600 µg of misoprostol was administered rectally. With the cessation of active bleeding, a control ultrasound confirmed the presence of chitosan gauze occupying the full uterine cavity, thus concluding the procedure.

**Figure 1:**
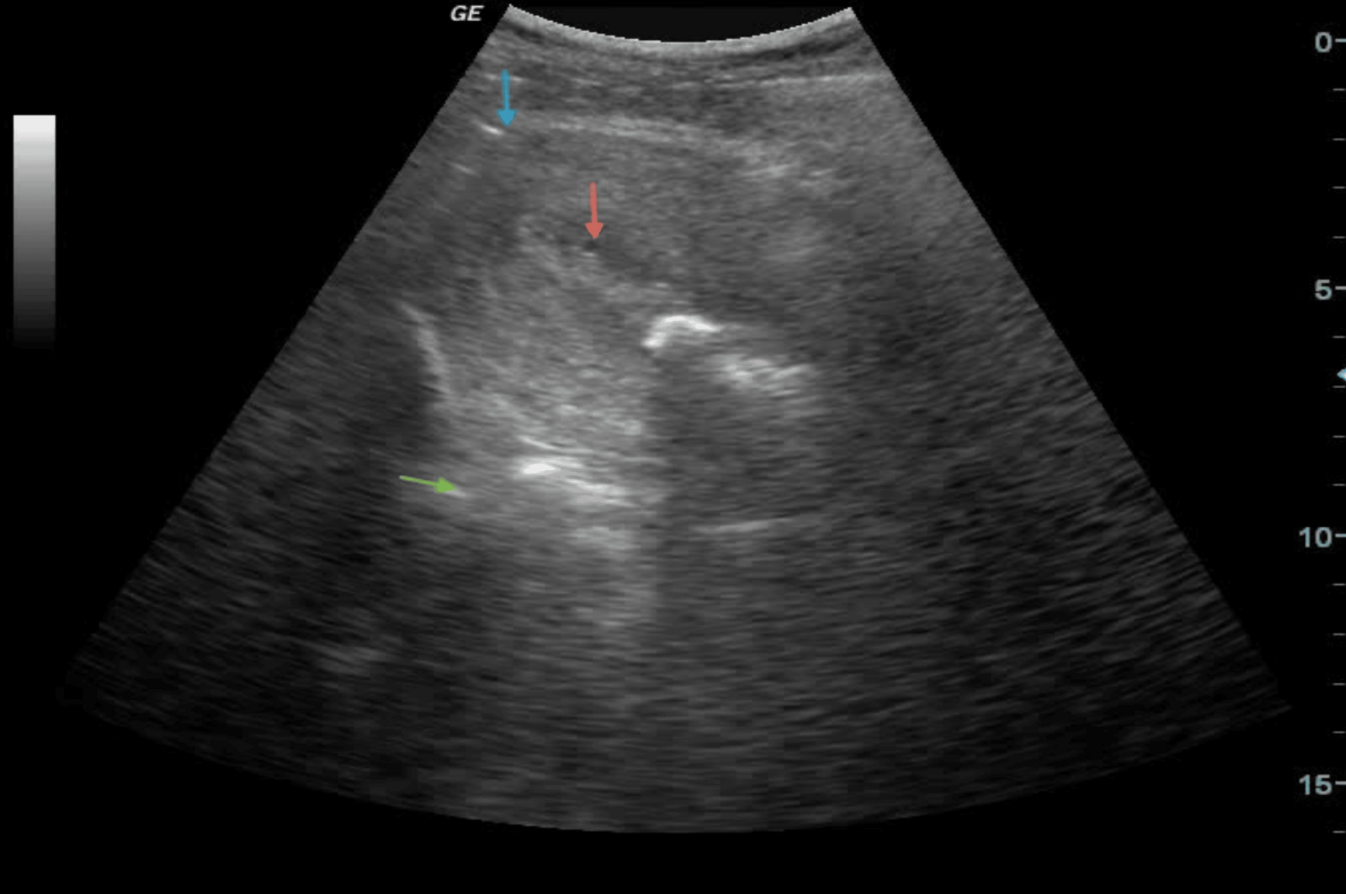
Abdominal ultrasound examination 18 hours post-delivery. blue arrow: uterine fundus; red arrow: chitosan gauze; green arrow: acoustic shadow

During the immediate recovery period and subsequent hours, there were no clinical signs of active transvaginal bleeding and no hemodynamic changes. An ultrasound examination, 18 hours post-delivery, visualized the chitosan gauze within the uterine cavity (Figure 1). 

**Figure 2:**
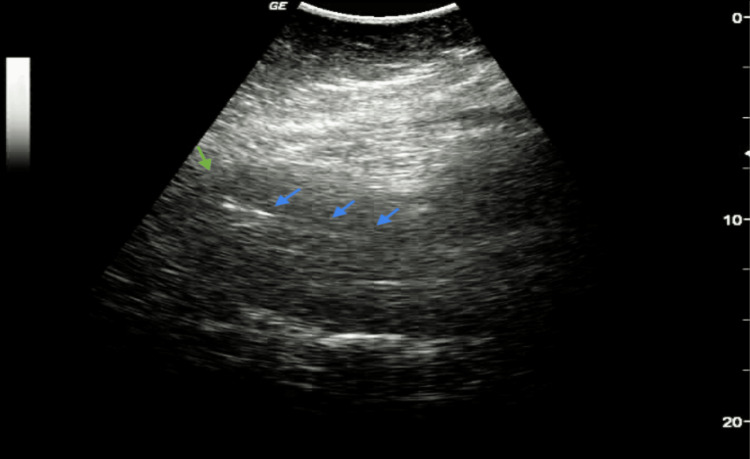
Abdominal control ultrasound. blue arrows: clean endometrial lining; green arrow: uterine fundus

In light of the patient’s normal vitals and absence of bleeding, we decided to extract the gauze, and an abdominal control ultrasound revealed a clean endometrial lining (Figure 2) without active transvaginal bleeding.

The patient was scheduled for an outpatient appointment seven days later. Vital signs were reported within normal parameters, without evidence of acute inflammatory syndrome. On physical examination, adequate uterine involution and absence of transvaginal bleeding were noted.

## Discussion

The first step in approaching a patient at risk of uterine bleeding is to identify the main risk factors, which are classified as low (single pregnancy, previous deliveries < 4, unscarred uterus, no history of PPH), medium (prior cesarean or uterine surgery, previous deliveries ≥ 4, multiple gestation, large uterine fibroids, chorioamnionitis, magnesium sulfate use, prolonged use of oxytocin), and high (placenta accreta spectrum, hematocrit < 30, bleeding at admission, known coagulation defect, history of PPH, tachycardia, and hypotension) risk [4]. 

**Figure 3:**
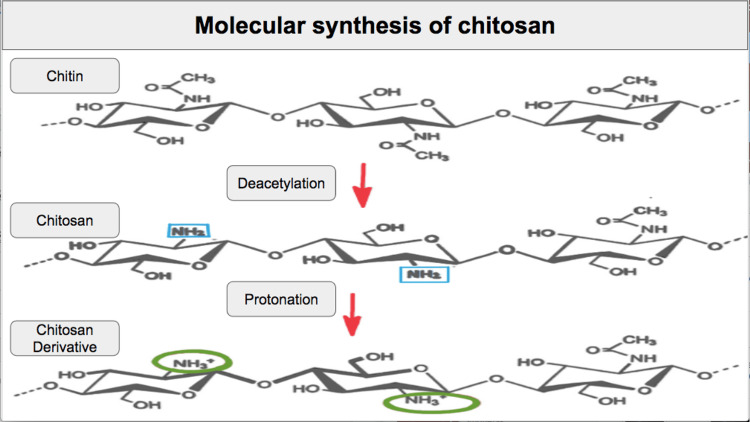
Molecular synthesis of chitosan. Image credit: Humberto López Maldonado

The mechanism of action of chitosan gauze is described in Figure 3.

Surgical techniques represent the final step in the treatment of PPH. The use of uterotonic drugs is recommended before considering surgical techniques, as the latter have a high morbidity. For example, the use of compressive sutures is associated with uterine necrosis and endometritis [12]. In addition, selective devascularization can cause fistula formation between arteria and vein, intraligamentary hematoma, and ureteral lesion [13].

Finally, the main complications of postpartum hysterectomy are bladder and ureteral injury, blood loss of 2-3 L, and a reported mortality of 1% [14].

Biele et al. [15] reported the use of chitosan gauze in a university hospital between May 2016 and May 2019. The study sample was divided into three groups (N=666): pharmacological management (n=530), Bakri balloon (n=55), and tamponade with chitosan gauze (n=85). The primary outcome, the need for a hysterectomy, was reduced by 77.8% with the introduction of chitosan gauze for the management of PPH.

Morbidity and mortality from PPH are higher in developing countries due to the lack of access to resources necessary for its treatment. As mentioned above, avoiding surgical treatments (e.g., compression sutures, and postpartum hysterectomy) could reduce morbidity in these patients and those in low-income countries. Mechanical measures to control PPH include the Bakri balloon, and in recent years chitosan gauze has been considered. The average cost of a Bakri balloon is 265 USD, and that of chitosan gauze is 63 USD [16], which makes the latter an excellent alternative for implementation in low-income countries.

Chitosan gauze has several advantages over the Bakri balloon, mainly its easier application, less probability of dislocation, and cheaper cost. On the other hand, Bakri ballon is approved by the FDA for PPH use; it also offers a major compression of the uterine vessels, and the presence of drainage provides easier quantification of bleeding [16]. 

In our case, the chitosan gauze was placed after the classic management of PPH failed. As the lithotomy position is recommended, control ultrasounds were performed in the delivery room and during hospitalization to verify the proper placement of the gauze.

The gauze was removed 24 hours after its placement. After the removal of the chitosan gauze, no more bleeding occurred. Gauze removal was performed painlessly, and complete withdrawal of the gauze was confirmed by transabdominal ultrasound.

**Figure 4:**
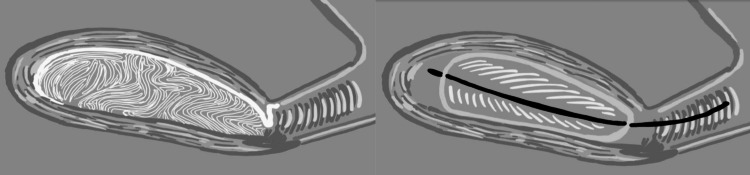
Schematic representation of chitosan gauze packing vs. Bakri balloon. One of the main advantages of chitosan gauze compared to the Bakri balloon is that it occupies the entire uterine cavity, leaving no space in the uterine fundus. Image credit: Humberto López Maldonado

After two days of hospitalization, the patient recovered well and was sent home two days later. Other treatment options for controlling PPH include O’Leary suture, clamping of uterine arteries with the Zea technique, uterine artery embolization, use of a Bakri balloon, or obstetric hysterectomy. Chitosan gauze seems to be superior to the Bakri balloon (Figure 4).

## Conclusions

Chitosan-coated gauze is a promising option for the treatment of PPH, as it reduces the postpartum hysterectomy rate without increased side effects compared to the Bakri balloon.

In addition, due to its lower cost compared to the Bakri balloon, it can be implemented in developing countries.

Easy gauze placement means that less training is required for its proper use. Therefore, hospitals lacking material resources or trained personnel for advanced PPH management can implement it, facilitating patient transfer to the closest secondary or tertiary hospital.
